# Association between maternal depressive symptoms during pregnancy and the risk of preeclampsia: a meta-analysis

**DOI:** 10.3389/fpsyt.2026.1764769

**Published:** 2026-03-16

**Authors:** Caixia Yi, Peiyu Cheng, Ning Peng, Yao Su, Huan Cao, Fangqun Cheng

**Affiliations:** 1 Department of Outpatient, Xiangtan Central Hospital, Xiangtan, China; 2 Hospital Party Committee Office, Xiangtan Central Hospital, Xiangtan, China; 3 Nursing Department, Xiangtan Central Hospital, Xiangtan, China

**Keywords:** depressive symptoms, meta-analysis, preeclampsia, pregnancy, risk factor

## Abstract

**Background:**

Depression during pregnancy is common and has been proposed as a potential risk factor for preeclampsia, but observational studies have reported inconsistent findings. This meta-analysis aimed to evaluate the association between maternal depressive symptoms during pregnancy and the subsequent risk of developing preeclampsia.

**Methods:**

PubMed, Embase, and Web of Science were systematically searched for observational studies evaluating depressive symptoms during pregnancy (measured before any assessment for preeclampsia) and reporting clinically diagnosed preeclampsia. Pooled odds ratios (ORs) and 95% confidence intervals (CIs) were calculated using a random-effects model accounting for the potential influence of heterogeneity.

**Results:**

Nine cohort studies involving 44,559 pregnant women, of whom 6,934 (15.6%) had depressive symptoms during pregnancy, were included. Maternal depressive symptoms was associated with a higher risk of preeclampsia (OR: 1.89; 95% CI: 1.17–3.03; *p* < 0.001; I² = 66%). The association was consistent across study design (prospective vs. retrospective), maternal age (< 29 vs. ≥ 29 years), and preeclampsia definition (hypertension plus proteinuria vs. hypertension plus organ involvement, *p* for subgroup difference all > 0.05). Stronger associations were observed in studies adjusting for parity (OR: 3.42 vs. 1.35; *p* for subgroup difference = 0.02). Adjustment for maternal body mass index attenuated the association (OR: 1.43 vs. 2.93; *p* for subgroup difference = 0.05).

**Conclusions:**

Depressive symptoms during early pregnancy is associated with an increased risk of subsequent preeclampsia. These findings highlight the importance of screening and monitoring depressive symptoms in routine prenatal care.

**Systematic Review Registration:**

PROSPERO, identifier CRD420251250417.

## Introduction

Preeclampsia is a pregnancy-specific multisystem disorder characterized by new-onset hypertension after 20 weeks of gestation accompanied by maternal–fetal organ dysfunction (1, 2). Traditionally defined by hypertension and proteinuria, diagnostic criteria have evolved in the past decade to include cases without proteinuria when evidence of systemic involvement is present, such as thrombocytopenia, renal or hepatic impairment, pulmonary edema, or new-onset neurological or visual symptoms (3). This broadened definition, now recognized by professional bodies including the American College of Obstetricians and Gynecologists (ACOG) and the International Society for the Study of Hypertension in Pregnancy (ISSHP), underscores the complex pathophysiology of the disease and its diverse clinical presentations (4). Affecting approximately 2–8% of pregnancies worldwide, preeclampsia remains a major contributor to maternal morbidity and mortality, particularly in low-resource settings (5). Complications include stroke, organ failure, placental abruption, and progression to eclampsia, while affected fetuses are at risk of prematurity, fetal growth restriction, and stillbirth (6, 7). Given its unpredictable clinical onset, improved early identification of women at risk is essential to facilitate targeted surveillance, preventive strategies, and timely obstetric management (8). Beyond traditional risk factors such as chronic hypertension, diabetes, obesity, and nulliparity, increasing attention has been directed toward potential psychophysiological determinants of preeclampsia (9).

Maternal depression, affecting an estimated 12–20% of pregnant women, has emerged as a plausible risk factor due to its biological interplay with immunologic dysfunction, endocrine dysregulation, abnormal placentation, heightened inflammation, oxidative stress, and vascular endothelial impairment—all central elements in the pathogenesis of preeclampsia (10–12). Although multiple epidemiological studies have examined this potential relationship, findings remain inconsistent, largely due to methodological limitations (13). Prior meta-analyses frequently did not differentiate the timing of depressive symptoms assessment, and several included studies in which depressive symptoms were assessed at or even after the diagnosis of preeclampsia, confounding temporal inference (14–16). Additionally, some studies relied on antidepressant prescription as the sole proxy for depressive symptoms, comparing users versus non-users rather than women with vs. without depressive symptoms, thereby reflecting treatment patterns rather than true mood status (17, 18). These weaknesses obscure whether depressive symptoms precede and contributes to preeclampsia risk. Therefore, an updated quantitative synthesis that incorporates contemporary diagnostic definitions of preeclampsia and restricts inclusion to studies assessing depressive symptoms before preeclampsia evaluation is warranted. The present meta-analysis was designed to clarify the temporal association between maternal depressive symptoms during pregnancy and the subsequent risk of preeclampsia based on validated exposure and outcome criteria.

## Methods

The conduct and reporting of this meta-analysis adhered to the PRISMA 2020 recommendations (19) and relevant guidance from the Cochrane Handbook (19), encompassing protocol development, data collection, statistical synthesis, and presentation of findings. The protocol was preregistered with PROSPERO (CRD420251250417).

### Database search

Eligible studies were located through an extensive literature search of PubMed, Embase, and Web of Science, employing a broad set of predefined search terms, which included (1): “pregnant” OR “pregnancy” OR “maternal” (2); “depression” OR “depressive” OR “mood” OR “affective disorder”; and (3) “preeclampsia” OR “pre-eclampsia” OR “pregnancy-induced hypertension” OR “hypertensive disorders of pregnancy” OR “gestational hypertension”. The search was restricted to human research and full-text articles published in English in peer-reviewed journals. To supplement the electronic search, reference lists of pertinent original studies and reviews were manually examined to identify additional eligible publications. All databases were searched from their inception through November 8, 2025. Detailed search strategies for each database are provided in **Supplementary File 1**.

### Study inclusion and exclusion

Study eligibility was defined according to the PICOS framework:

#### P (population)

Pregnant women of any maternal age, ethnicity, parity, or socioeconomic background.

#### I (exposure)

Maternal depressive symptoms during pregnancy, assessed before any diagnosis or evaluation of preeclampsia, defined consistent with the criteria used in the original studies. In general, maternal depressive symptoms could be defined by clinical diagnosis based on standardized criteria such as Diagnostic and Statistical Manual of Mental Disorders (DSM) or International Classification of Disease (ICD) codes, depressive symptoms measured using validated tools with specified cut-offs, or prescription/use of antidepressants only when clearly used as a proxy for depressive symptoms during pregnancy, and when depressive symptoms or diagnosis are reported or inferable.

#### Comparator (C)

Pregnant women without depressive symptoms during pregnancy, defined by clinical assessment, validated screening below threshold, or explicitly stated absence of depressive symptoms, but not simply by lack of antidepressant use alone.

#### Outcomes (O)

Clinically diagnosed preeclampsia, according to established diagnostic criteria which are consistent with those in the original studies, occurring after exposure assessment.

#### S (Study design)

Observational designs capable of establishing exposure–outcome temporality, which include prospective or retrospective cohort studies, case-control and nested case-control studies, and population-based registry studies.

Studies were excluded if they lack clear temporal assessment of maternal depressive symptoms prior to preeclampsia diagnosis, including research measuring depressive symptoms only postpartum, reporting lifetime or prepregnancy depressive symptoms without reassessment during pregnancy, or failing to specify the timing of depressive symptoms evaluation. Studies that use antidepressant prescriptions as the sole indicator of depressive symptoms were excluded unless depressive symptoms or clinical diagnosis during pregnancy were reported or reasonably inferable. Articles that did not define preeclampsia clearly or combine preeclampsia with other hypertensive disorders without stratified data were also excluded. Cross-sectional designs, reviews, commentaries, clinical trials, animal studies, and case reports were also excluded. If multiple articles were derived from the same underlying cohort, we included only the version with the most extensive dataset or the largest cohort.

### Study quality evaluation

Two reviewers independently performed the literature search, study screening, quality appraisal, and data extraction, with any disagreements resolved through discussion and consensus between with the authors. Study quality was assessed using the Newcastle–Ottawa Scale (NOS) (20), which evaluates selection, adjustment for confounding, and outcome assessment, yielding scores from 1 to 9, with higher scores indicating better methodological rigor. Studies scoring ≥ 7 were classified as high quality.

### Data collection

Extracted data included study-level information (author, publication year, country, and design), patient characteristics (sample size, and mean maternal age), exposure characteristics (timing for the evaluation of depressive symptoms, methods for the diagnosis of depressive symptoms, and numbers of women with depressive symptoms during pregnancy), outcome characteristics (diagnostic criteria for preeclampsia, and numbers of women who developed preeclampsia in each study), and covariates adjusted for in the estimation of the association between maternal depressive symptoms and preeclampsia risk.

### Statistical analysis

The influence of maternal depressive symptoms during pregnancy on risk of preeclampsia was summarized as odds ratio (OR) and corresponding 95% confidence intervals (CIs), compared between women with vs. without depressive symptoms during pregnancy. When necessary, ORs and standard errors were derived from reported CIs or *p*-values and subsequently log-transformed to stabilize variance and approximate normality (19). Between-study heterogeneity was examined using the Cochrane Q statistic and the I² metric (21), with thresholds of < 25%, 25–75%, and > 75% interpreted as low, moderate, and high heterogeneity, respectively. Pooled estimates were generated using a random-effects model to account for underlying variability across studies (19). Robustness of the overall effect was evaluated through leave-one-out sensitivity analyses (22). Prespecified subgroup analyses were performed to explore whether study characteristics influenced the observed associations. Stratifications included study design (prospective vs. retrospective), mean maternal ages, definition of preeclampsia (hypertension and proteinuria vs. hypertension and organ involvement), adjustment of parity, adjustment of maternal body mass index (BMI), and NOS scores. For subgroup analyses involving continuous study-level variables, including mean maternal age, cutoff values were defined according to the median of the reported study-level means to ensure a balanced distribution of studies across subgroups and to preserve analytical stability in the absence of individual participant data. Potential publication bias was evaluated using funnel plot symmetry, visual inspection, and Egger’s regression test (23). A two-sided *p*-value < 0.05 was considered statistically significant. All analyses were performed using RevMan (version 5.3; Cochrane Collaboration, Oxford, UK) and Stata (version 17.0; StataCorp, College Station, TX, USA).

## Results

### Study inclusion

**Figure 1:**
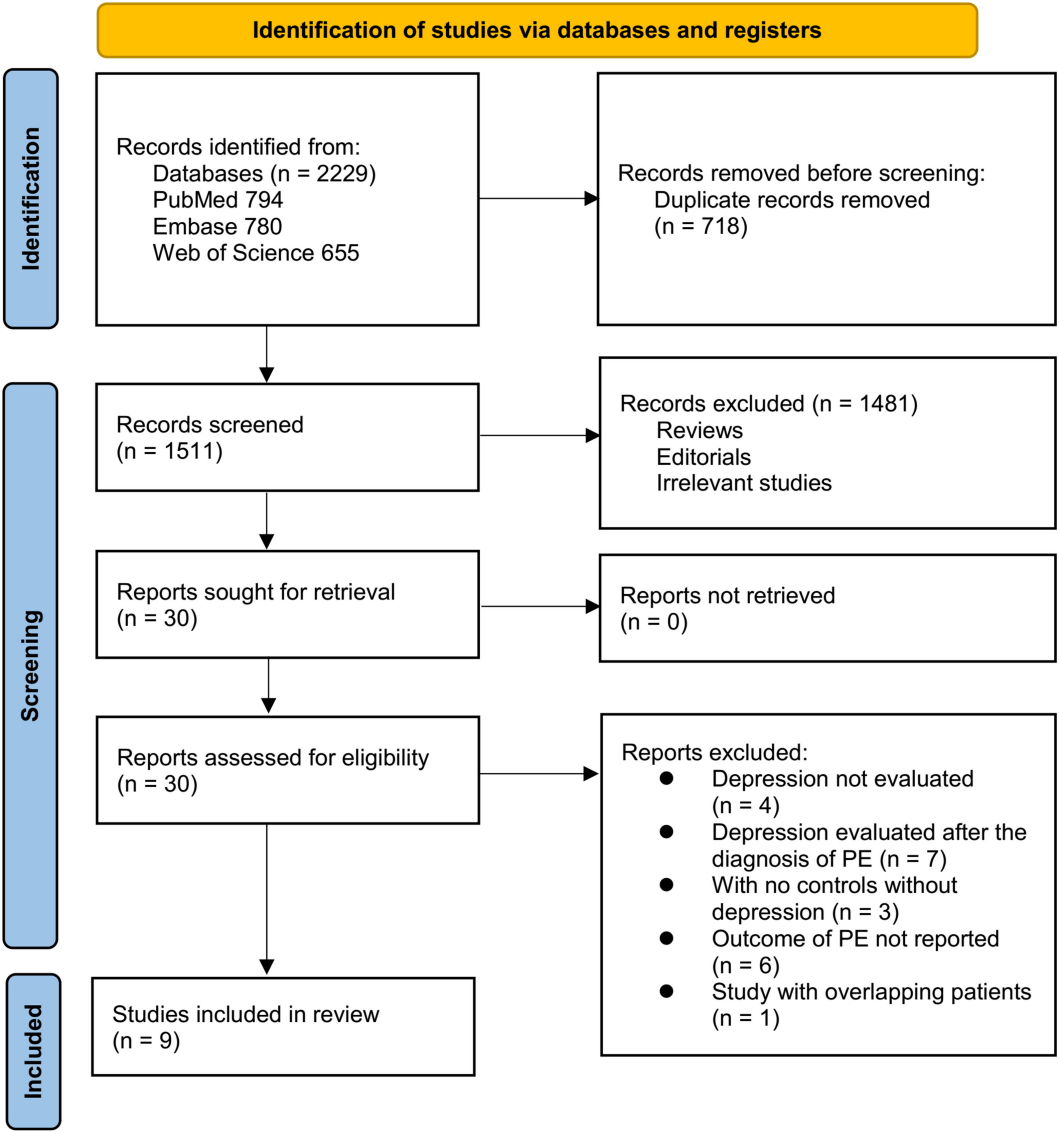
Flowchart of database search and study inclusion.

**Figure 1** depicts the study selection workflow. A total of 2,229 records were retrieved from the three databases, of which 718 duplicates were removed. Screening of titles and abstracts resulted in the exclusion of 1,481 records that did not meet the eligibility criteria. The full texts of the remaining 30 articles were evaluated independently by two reviewers, and 21 were excluded for reasons shown in **Figure 1**. Ultimately, nine studies met all criteria and were included in the quantitative synthesis (24–32).

### Overview of the study characteristics

The characteristics of the included studies are summarized in **Table 1**. A total of nine cohort studies (24–32) published between 2000 and 2025 were included, representing populations from Finland, Peru, the Netherlands, the United States, Canada, and Australia. Of these, six were prospective studies (24, 26, 29–32) and three were retrospective (25, 27, 28), with sample sizes ranging from 261 to 21,589 women, yielding a combined population of 44,559 pregnant women. The mean maternal age ranged from 24.6 to 32.0 years, and all studies evaluated depressive symptoms before 20 weeks of gestation, ensuring that depressive symptoms were assessed prior to the development of preeclampsia. Depressive symptoms were diagnosed using validated screening tools or clinical assessment, including the Beck Depression Inventory (BDI) (24), Patient Health Questionnaire-9 (PHQ-9) (25, 28), Center for Epidemiologic Studies Depression Scale (CES-D) (26, 29), Edinburgh Postnatal Depression Scale (EPDS) (27, 32), and the DSM-IV Structured Clinical Interview (SCID-IV) (31), or evidenced via medical records (30). In total, 6,934 women (15.6%) were diagnosed with depressive symptoms during pregnancy. Preeclampsia diagnosis consistently required new-onset hypertension after 20 weeks of gestation accompanied by proteinuria (24–30) or organ involvement (31, 32). Overall, 1,722 (3.9%) women developed preeclampsia during pregnancy. In terms of statistical adjustment, eight studies (24–31) controlled for important confounding variables, including maternal age, BMI, ethnicity, parity, smoking, socioeconomic status, and medical comorbidities to a varying degree, while one large cohort did not include adjustment (32) in estimating the association between depressive symptoms and preeclampsia.

**Table 1:** Characteristics of the included studies.

Study	Country	Design	No. of women	Mean age (years)	Timing for the diagnosis of depressive symptoms	Methods for the diagnosis of depressive symptoms	No. of women with depressive symptoms	Diagnostic criteria for PE	No. of women with PE	Variables adjusted
Kurki 2000 (24)	Finland	PC	623	NR	Median GA 12 weeks (range 10–17 weeks)	Finnish modification of BDI ≥ 3	185	BP > 140/100 mmHg + proteinuria ≥ 0.3g/24h after 20 weeks’ gestation	28	Maternal age, smoking, alcohol consumption, marital status, socioeconomic status, and bacterial vaginosis
Qiu 2007 (25)	Peru	RC	676	26.3	Before the GA of 20 weeks	PHQ-9 ≥ 10	76	Sustained BP ≥ 140/90 mmHg + proteinuria ≥ 30 mg/dl (or 1+ dipstick) after 20 weeks	339	Maternal age, nulliparity, and pre-pregnancy overweight (BMI > 26.0 kg/m²)
Vollebregt 2008 (26)	The Netherlands	PC	3679	29.9	Median GA 15.6 weeks (quartiles 14.0-17.3 weeks)	CES-D, Dutch version; categories: > 90th	368	BP ≥ 140/90 mmHg + proteinuria ≥ 0.3g/24h or dipstick ≥ ++ after 20 weeks	128	Maternal age, ethnicity, education, marital status, BMI, chronic hypertension, diabetes, smoking, previous miscarriage/abortion, and vaginal hemorrhage
Kim 2013 (27)	USA	RC	261	24.6	Mean 17.2 weeks (Before the GA of 20 weeks)	EPDS ≥ 10	91	Hypertension + proteinuria per medical record (ACCG/ISSHP criteria implied)	25	Maternal age, and parity
Avalos 2015 (28)	USA	RC	21589	NR	Before the GA of 20 weeks	PHQ-9 ≥ 10	5038	ICD-9 codes after 20 weeks gestation	972	Maternal age, BMI, race/ethnicity, marital status, parity, alcohol use, smoking, diabetes, and other mental health diagnoses
Thombre 2015 (29)	USA	PC	1371	26.3	Before the GA of 20 weeks	CES-D ≥ 16	62	Hypertension + proteinuria (≥ 300 mg/24h or 1+ dipstick twice after 20 weeks)	44	Maternal age, race/ethnicity, Medicaid status, smoking history, prepregnancy BMI
Bernard 2019 (30)	Canada	PC	6761	29.9	Before the GA of 16 weeks	Medical records evidenced	259	Hypertension + proteinuria (≥ 300 mg/24h or ≥ 2+ dipstick)	122	Maternal age, pre-pregnancy BMI, pre-pregnancy hypertension, ethnicity, parity, smoking during pregnancy, MAP at first visit, past history of HDP, and GDM
Galbally 2023 (31)	Australia	PC	815	32.0	Early pregnancy (< 20 weeks of GA)	DSM-IV (SCID-IV)	229	Hypertension after 20 weeks + organ involvement	21	Maternal age, obesity (BMI ≥ 30), parity, smoking during pregnancy, university education, and relationship status
Bank 2025 (32)	USA	PC	8784	27.0	Early pregnancy (< 6-13 weeks of GA)	EPDS ≥ 13	626	Hypertension after 20 weeks + organ involvement	43	None

NR, not reported; PC, prospective cohort; RC, retrospective cohort; GA, gestational age; BDI, Beck Depression Inventory; PHQ-9, Patient Health Questionnaire-9; CES-D, Center for Epidemiologic Studies Depression Scale; EPDS, Edinburgh Postnatal Depression Scale; DSM-IV, Diagnostic and Statistical Manual of Mental Disorders, Fourth Edition; SCID-IV, Structured Clinical Interview for DSM-IV; BP, blood pressure; BMI, body mass index; ICD-9, International Classification of Diseases, Ninth Revision; ACCG/ISSHP, American College of Obstetricians and Gynecologists/International Society for the Study of Hypertension in Pregnancy; MAP, mean arterial pressure; HDP, hypertensive disorders of pregnancy; GDM, gestational diabetes mellitus.

### Study quality evaluation

Study quality was assessed using the NOS, as shown in **Table 2**. Total NOS scores ranged from 7 to 9, indicating that the overall methodological quality of the included studies was moderate to high. Five studies (24, 26, 29–31) achieved a score of 9, characterized by strong cohort representativeness, validated depressive symptoms assessment, appropriate confirmation that preeclampsia was absent at baseline, adequate adjustment for key confounders, standardized assessment of preeclampsia, and sufficient follow-up duration with complete outcome ascertainment. Three studies scored 8, generally reflecting minor limitations in representativeness (27, 28) or ascertainment of exposure (25), despite otherwise strong methodology. One study (32) scored 7, primarily because it did not adjust for confounding variables, although it met other cohort and outcome assessment criteria. Importantly, no study was rated as poor quality, and all met core requirements for valid cohort design, proper temporal assessment of exposure and outcome, and reliable diagnostic measurement. These quality assessments support a robust and credible evidence base for evaluating the association between depressive symptoms during pregnancy and the risk of developing preeclampsia.

**Table 2:** Study quality evaluation via the Newcastle-Ottawa scale.

Study	Representativeness of the exposed cohort	Selection of the non-exposed cohort	Ascertainment of exposure	Outcome not present at baseline	Control for age	Control for other confounding factors	Assessment of outcome	Enough long follow-up duration	Adequacy of follow-up of cohorts	Total
Kurki 2000 (24)	1	1	1	1	1	1	1	1	1	9
Qiu 2007 (25)	1	1	0	1	1	1	1	1	1	8
Vollebregt 2008 (26)	1	1	1	1	1	1	1	1	1	9
Kim 2013 (27)	0	1	1	1	1	1	1	1	1	8
Avalos 2015 (28)	0	1	1	1	1	1	1	1	1	8
Thombre 2015 (29)	1	1	1	1	1	1	1	1	1	9
Bernard 2019 (30)	1	1	1	1	1	1	1	1	1	9
Galbally 2023 (31)	1	1	1	1	1	1	1	1	1	9
Bank 2025 (32)	1	1	1	1	0	0	1	1	1	7

### Association between maternal depressive symptoms and preeclampsia risk

**Figure 2:**
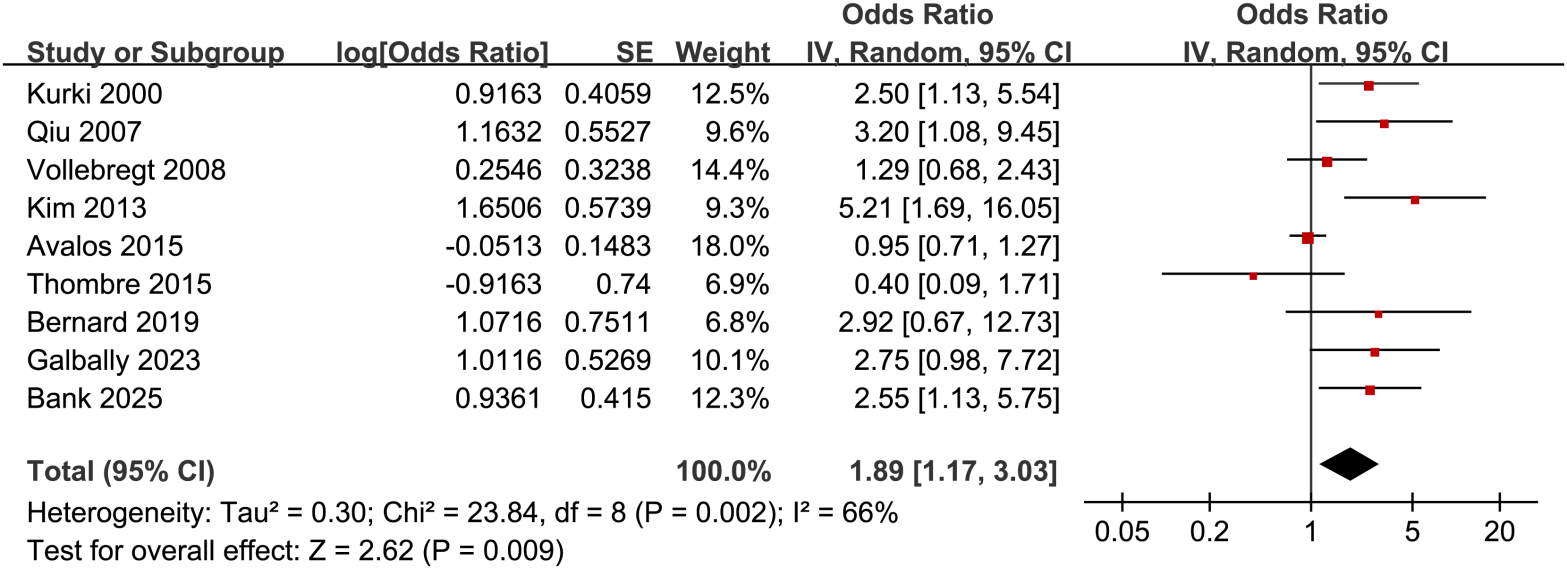
Forest plots for the meta-analysis of the association between maternal depressive symptoms during pregnancy and the risk of preeclampsia.

**Figure 3:**
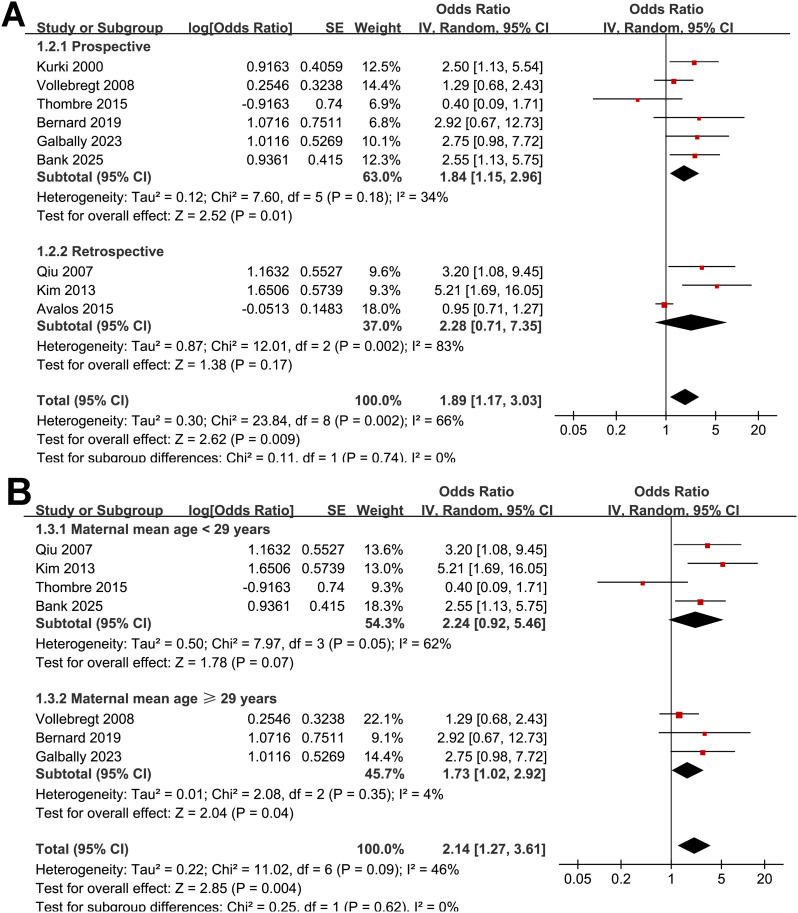
Forest plots for the subgroup analysis of the association between maternal depressive symptoms during pregnancy and the risk of preeclampsia. **(A)** subgroup analysis according to study design; and **(B)** subgroup analysis according to mean maternal age.

**Figure 4:**
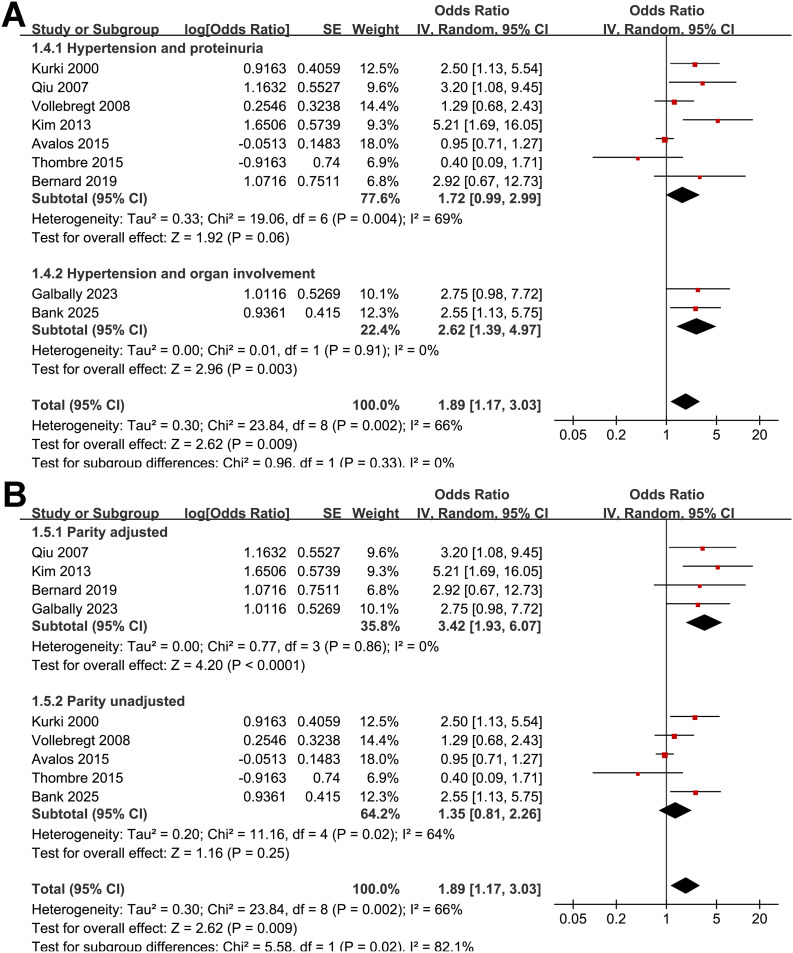
Forest plots for the subgroup analysis of the association between maternal depressive symptoms during pregnancy and the risk of preeclampsia. **(A)** subgroup analysis according to diagnostic criteria of preeclampsia; and **(B)** subgroup analysis according to the adjustment of parity.

**Figure 5:**
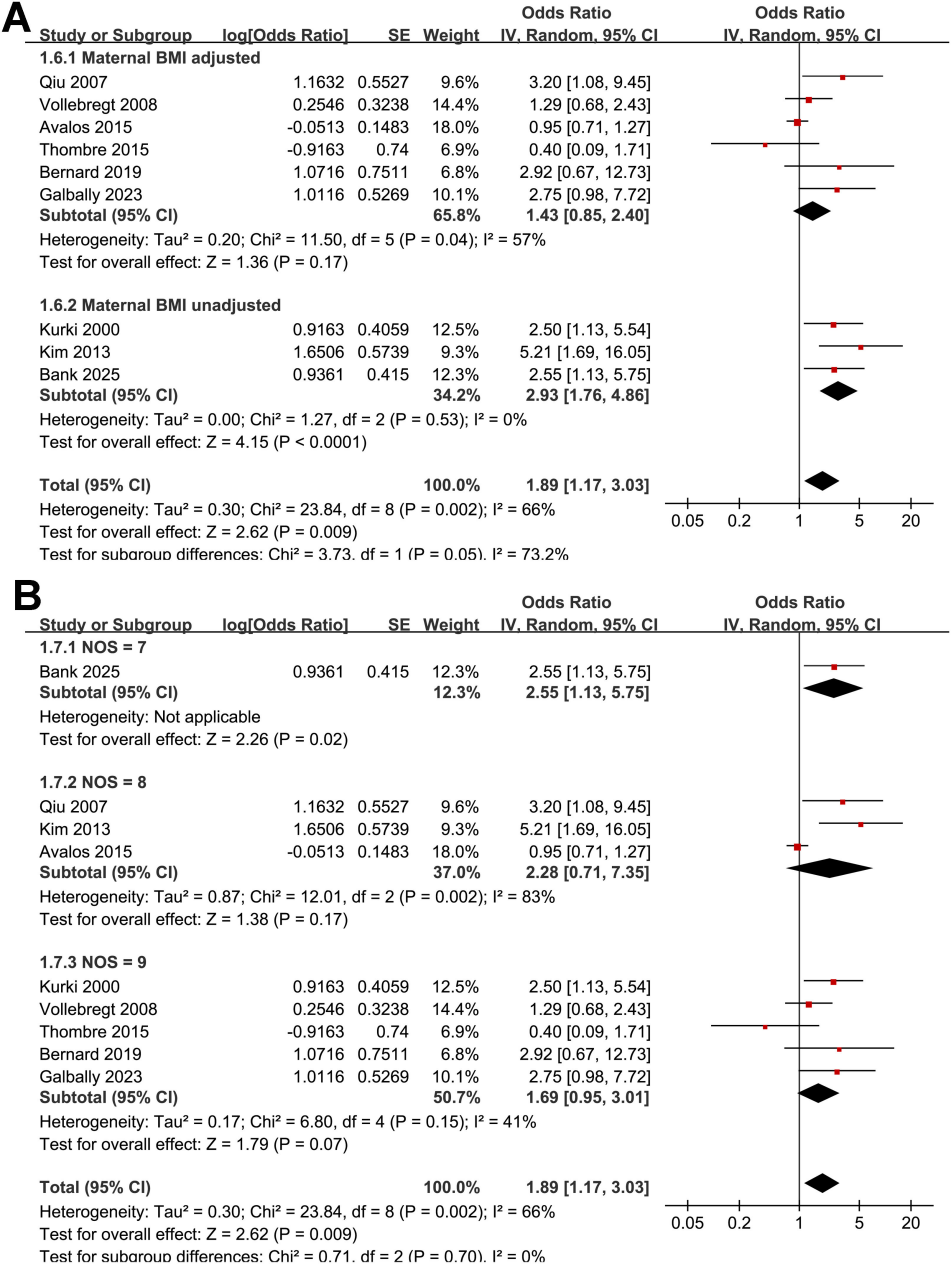
Forest plots for the subgroup analysis of the association between maternal depressive symptoms during pregnancy and the risk of preeclampsia. **(A)** subgroup analysis according to the adjustment of maternal BMI; and **(B)** subgroup analysis according to the NOS scores;.

Pooled results of the nine studies (24–32) showed that compared to women without depressive symptoms during pregnancy, those with depressive symptoms in pregnancy were associated with an increased risk of preeclampsia (OR: 1.89, 95% CI: 1.17 to 3.03, *p* < 0.001; **Figure 2**) with moderate heterogeneity (*p* for Cochrane Q test = 0.002; I^2^ = 66%). Sensitivity analysis by excluding one study at a time showed consistent results (OR: 1.68 to 2.18, *p* all < 0.05). Further subgroup analyses demonstrated consistent associations between depressive symptoms during pregnancy and preeclampsia across multiple study-level characteristics. Similar effect estimates were observed between prospective and retrospective cohorts (OR: 1.84 vs. 2.28; *p* for subgroup difference = 0.74; **Figure 3A**), between studies with a mean maternal age < 29 versus ≥ 29 years (OR: 2.24 vs. 1.73; *p* = 0.62; **Figure 3B**), and between studies defining preeclampsia as hypertension plus proteinuria versus hypertension plus organ involvement (OR: 1.72 vs. 2.62; *p* = 0.33; **Figure 4A**). Notably, studies that adjusted for parity reported a markedly stronger association than those without such adjustment (OR: 3.42 vs. 1.35; *p* = 0.02; **Figure 4B**). Additionally, the association was attenuated when maternal BMI was adjusted for (OR: 1.43 vs. 2.93), although the difference did not reach statistical significance (*p* = 0.05; **Figure 5A**). Consistent findings were also observed across studies with NOS scores of 7, 8, and 9 (OR: 2.55, 2.28, and 1.69, respectively; *p* for subgroup difference = 0.70; **Figure 5B**).

### Publication bias

**Figure 6:**
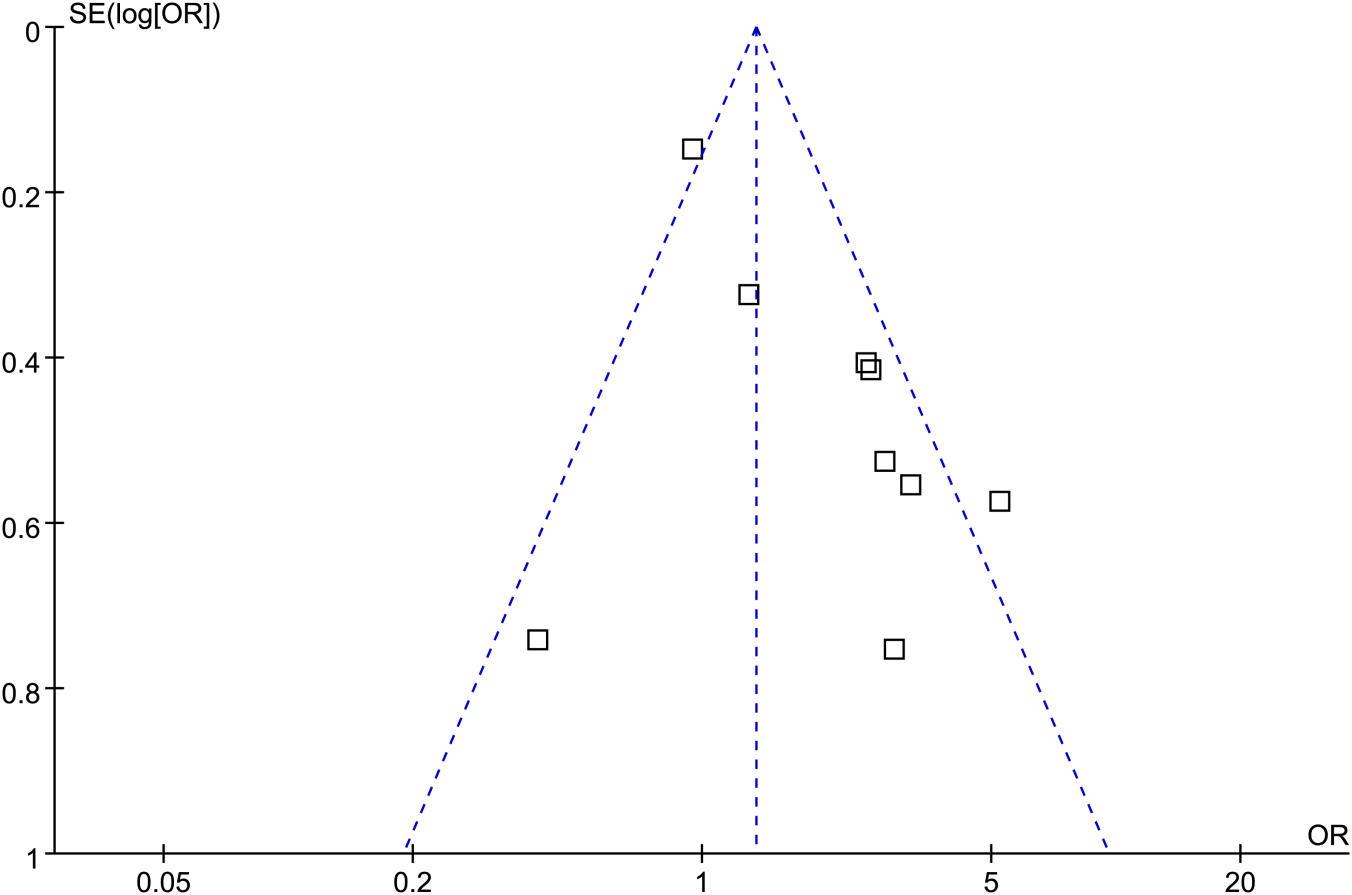
Funnel plots estimating the potential publication bias underlying the meta-analysis of the association between maternal depressive symptoms during pregnancy and the risk of preeclampsia.

**Figure 6** displays the funnel plots evaluating potential publication bias for the meta-analyses of the association between maternal depressive symptoms during pregnancy and the risk of preeclampsia. No obvious asymmetry was observed on inspection, and Egger’s regression tests did not suggest significant small-study effects (*p* = 0.34).

## Discussion

The findings of this meta-analysis indicate that depressive symptoms diagnosed during early pregnancy is associated with an elevated risk of subsequently developing preeclampsia. Importantly, the association persisted across diverse study designs, maternal characteristics, and diagnostic definitions of preeclampsia, and remained stable in sensitivity analyses, strengthening confidence that the observed relationship is unlikely to be explained solely by chance or study heterogeneity. These results support the hypothesis that mood disturbances occurring before the clinical onset of preeclampsia may contribute to the pathophysiological processes leading to the disorder, rather than simply co-occurring as a consequence of adverse pregnancy outcomes. Given that preeclampsia typically manifests after mid-gestation but originates from placental and systemic abnormalities that begin much earlier, the presence of depressive symptoms in the first half of pregnancy may coincide with, or even influence, the early disease-development window in susceptible women.

Several biological mechanisms may underpin the link between maternal depressive symptoms and preeclampsia. Depression is characterized by dysregulation of the hypothalamic–pituitary–adrenal axis, resulting in elevated corticotropin-releasing hormone and cortisol levels, both of which influence placental development and endothelial adaptation (33–35). Dysregulated immune activation, including heightened pro-inflammatory cytokines such as interleukin-6 and tumor necrosis factor-alpha, has been documented in depressive symptoms and may contribute to abnormal trophoblast invasion, oxidative stress, and endothelial dysfunction—all crucial features of preeclampsia (36–38). Pregnant women with depression may also exhibit altered autonomic balance, hypercoagulability, reduced nitric oxide availability, and impaired vascular reactivity, further supporting a shared mechanistic pathway with hypertensive disorders of pregnancy (39, 40). Although the current evidence does not clarify whether depressive symptoms alone are causative or interacts with coexisting maternal conditions, these biological pathways provide a plausible explanation for the observed association.

The subgroup results offer additional insight into potential mechanisms and suggest that certain maternal characteristics may modify the strength of association. A notably stronger association was observed in studies adjusting for parity, which is a well-known risk factor for preeclampsia. One possible explanation is that nulliparous women, who are inherently at higher risk of preeclampsia, may experience compounded vulnerability when depressive symptoms are present, perhaps due to exaggerated autonomic, hormonal, or inflammatory responses in a first pregnancy (41, 42). Conversely, the association was attenuated when maternal BMI was accounted for. Obesity is strongly linked to both depressive symptoms and preeclampsia, through shared inflammatory, endocrine, and metabolic pathways (43, 44). Adjustment for BMI may therefore reduce confounding by a shared mechanistic substrate rather than revealing a weaker causal effect. The attenuation toward null after BMI adjustment should not be interpreted as negating the association, but rather suggests that mood disturbances and metabolic/inflammatory dysregulation may intersect in the development of preeclampsia. Another notable finding was the lack of significant subgroup differences between older and younger maternal age, suggesting that depressive symptoms may be a risk factor across age strata, unlike many traditional risk factors that disproportionately affect women at later reproductive ages.

Although the overall pooled estimate suggested an increased risk of preeclampsia among women with depressive symptoms during pregnancy, notable variability existed across individual studies. Five of the included studies reported non-significant associations (26, 28–31), and two of them (28, 29) demonstrated effect estimates below unity, underscoring the inconsistency of the existing evidence. This heterogeneity constituted a major rationale for undertaking the present meta-analysis. Prespecified subgroup analyses identified potential modifying factors, including adjustment for parity and maternal BMI. However, these analyses did not fully account for the observed between-study differences. Such inconsistency may reflect variations in depressive symptoms assessment tools, diagnostic thresholds, timing of exposure measurement, population characteristics, and residual or unmeasured confounding. Owing to the absence of individual participant data, the underlying causes of heterogeneity could not be entirely delineated and should be considered when interpreting the findings.

Interpretation of these results must also consider the strengths of the current meta-analysis. First, this study applied strict inclusion criteria, limiting the analysis to studies assessing depressive symptoms prior to any clinical evaluation for preeclampsia, thereby establishing a clearer temporal sequence than previous meta-analyses (14–16). Second, a comprehensive and up-to-date search strategy across multiple databases captured recent evidence from geographically diverse populations. Third, multiple subgroup and sensitivity analyses were conducted, consistently demonstrating that the association was robust to variations in study methodology, population characteristics, and definitions of preeclampsia. Finally, the diagnostic evolution of preeclampsia was incorporated into the analysis, recognizing that not all cases require proteinuria under contemporary criteria, and enabling unified interpretation within current clinical frameworks. Nonetheless, several limitations should be acknowledged. Some included studies were retrospective cohorts, in which reliance on medical records or administrative coding may introduce misclassification or incomplete ascertainment of depressive symptoms and preeclampsia. Variability in measurement tools, diagnostic cutoffs for depressive symptoms, and clinician-driven diagnosis or coding of preeclampsia may have contributed to heterogeneity that could not be fully explored due to the absence of individual participant data. Although most studies adjusted for multiple confounders, unmeasured or residual confounding cannot be excluded, particularly regarding psychosocial stress, sleep disorders, substance use, socioeconomic disadvantage, or comorbid anxiety, which frequently co-occur with depressive symptoms and may influence preeclampsia risk. Publication bias remains possible despite non-significant Egger’s test results, as studies with null findings may be less likely to be published. Finally, observational studies can identify temporal and statistical associations, but cannot establish causation, and the present results should be interpreted as evidence of association rather than proof of a causal relationship.

From a clinical perspective, these findings suggest that systematic screening for depressive symptoms during early pregnancy—already recommended in many countries for maternal mental health (45)—may also contribute to risk stratification for preeclampsia. Women with significant depressive symptoms could benefit from enhanced monitoring of blood pressure and clinical markers, early lifestyle or psychological interventions, and closer surveillance for preeclampsia-related symptoms. However, routine implementation of antidepressants solely for preeclampsia prevention is not justified based on current evidence, and concerns regarding medication effects, dose, and timing must be weighed carefully (46). Future research should prioritize prospective cohort studies with standardized depressive symptoms assessment tools, incorporation of biological biomarkers, evaluation of dose–response relationships, and separate analyses of treated versus untreated depressive symptoms to clarify whether improved mental health management modifies preeclampsia risk.

## Conclusions

In conclusion, depressive symptoms during early pregnancy is associated with an increased risk of developing preeclampsia later in gestation. These findings reinforce the clinical importance of early detection and management of depressive symptoms as part of comprehensive prenatal care. Further investigations are warranted to elucidate causal pathways and to determine whether targeted psychosocial or medical interventions can reduce the occurrence of preeclampsia among at-risk women.

## Data Availability

The original contributions presented in the study are included in the article/**Supplementary Material**. Further inquiries can be directed to the corresponding author.
