# Supplementary material for: Association between maternal depressive symptoms during pregnancy and the risk of preeclampsia: a meta-analysis
Source: Front Psychiatry. 2026 Mar 16;17:1764769. doi: 10.3389/fpsyt.2026.1764769 (PMC13033700; doi:10.3389/fpsyt.2026.1764769)
Supplement: Supplementary file 1 [file Table1.docx]

**Detailed search strategy for each database**

**PubMed**

("Pregnancy"[Mesh] OR pregnant*[tiab] OR pregnanc*[tiab] OR maternal[tiab]) AND ("Depression"[Mesh] OR "Depressive Disorder"[Mesh] OR depression[tiab] OR depressive[tiab] OR "affective disorder*"[tiab] OR "mood disorder*"[tiab]) AND

("Preeclampsia"[Mesh] OR "Hypertension, Pregnancy-Induced"[Mesh] OR preeclampsia[tiab] OR "pre-eclampsia"[tiab] OR "gestational hypertension"[tiab] OR "pregnancy-induced hypertension"[tiab] OR "hypertensive disorders of pregnancy"[tiab])

**Embase**

('pregnancy'/exp OR pregnant*:ti,ab OR pregnanc*:ti,ab OR maternal:ti,ab) AND ('depression'/exp OR 'depressive disorder'/exp OR depression:ti,ab OR depressive:ti,ab OR 'affective disorder*':ti,ab OR 'mood disorder*':ti,ab) AND ('preeclampsia'/exp OR 'pregnancy induced hypertension'/exp OR preeclampsia:ti,ab OR 'pre-eclampsia':ti,ab OR 'gestational hypertension':ti,ab OR 'pregnancy-induced hypertension':ti,ab OR 'hypertensive disorders of pregnancy':ti,ab) NOT (animal/exp NOT human/exp)

**Web of Science**

TS = ((pregnant* OR pregnanc* OR maternal) AND (depression OR depressive OR "affective disorder*" OR "mood disorder*") AND (preeclampsia OR "pre-eclampsia" OR "gestational hypertension" OR "pregnancy-induced hypertension" OR "hypertensive disorders of pregnancy"))
